# Polypropylene Suture Guided Microdochectomy for Pathologic Nipple Discharge

**DOI:** 10.4274/balkanmedj.2017.1319

**Published:** 2018-07-24

**Authors:** Emine Özlem Gür, Serkan Karaisli, Akif Serhat Gür, Mehmet Hacıyanlı

**Affiliations:** 1 Clinic of General Surgery, İzmir Katip Çelebi University, Atatürk Training and Research Hospital, İzmir, Turkey; 2 Clinic of General Surgery, Private Tınaztepe Hospital, İzmir, Turkey

To the Editor,

Nipple discharge is a complaint of approximately 5% of women. Pathologic nipple discharge is defined as unilateral, spontaneous discharge from a single duct during the nonlactational period. Benign lesions such as intraductal papilloma and mammary duct ectasia are the reasons for pathologic nipple discharge. The association between pathologic nipple discharge and malignancy is approximately 10%-20% (1). Bloody nipple discharge is considered as highly suspicious for malignancy or ductal carcinoma *in situ* of the breast (2). Patients with pathologic nipple discharge should be evaluated to rule out malignancy.

A total of 78 patients were admitted to our clinic with a complaint of nipple discharge between January 2011 and January 2017. Physical examination, ultrasound, and mammography (for patients older than 40 years) were performed. Magnetic resonance imaging was applied to patients with no pathological findings on the mammography and/or the ultrasound. Five patients who had no pathological findings on ultrasound, mammography, or magnetic resonance imaging underwent polypropylene suture guided microdochectomy.

Patients were prepared under general anesthesia in the operating room in the supine position. The discharging quadrant was determined by physical examination before incision. Surgipro Monofilament polypropylene 2-0 (Covidien, Dublin, Ireland) was inserted into the ductus via the orifice (Figure 1a). The polypropylene became palpable, and the blue color became noticeable in the breast tissue after the circumareolar incision (Figure 1b). The bloody discharging duct was determined with certainty after observing the polypropylene after a mini incision on the discharging duct (Figure 1c). The guided ductus was excised with the normal margin of the breast tissue. The incision was closed in the anatomical planes.

The pathological examination showed that one patient had ductal carcinoma *in situ* (20.0%), two had intraductal papilloma, and two had cystic disease of the breast.

All patients were followed up during regular intervals of 3 months. After 1-year follow-up, there was no bloody nipple discharge recurrence. The patient with ductal carcinoma *in situ* underwent radiation therapy. There was no local or systemic recurrence after the radiation therapy in the 1-year follow-up. Informed consents were obtained from all the patients. This study was approved by the ethics committee of our hospital.

Physical examination is the first approach to evaluate the disorder. The aim of physical examination is to detect the discharging quadrant of the breast and the ductal orifice. Once the discharging orifice is detected, the next step is the imaging techniques. If there are only clinical findings but no imaging findings, several diagnostic and treatment methods can be performed to detect the pathologic duct. Marking with blue dye staining (3) and wire localization under galactography guidance (4) are the primary preoperative methods. In addition to these methods, intraoperative intraductal blue dye injection, ductoscopic wire marking (5), and insertion of a lacrimal probe into the discharging duct (2) can be performed. 

Dillon et al. (2) reported a recurrence rate of 9% during 1-year follow-up and a median recurrence time of 7 months after microdochectomy in their study. None of our patients had recurrent disease in the 1-year follow-up.

In conclusion, polypropylene suture guided microdochectomy does not require additional duct-specific imaging methods to determine and resect the pathologic discharging duct. Polypropylene suture guided microdochectomy is an inexpensive, useful, easy, and reliable method to treat patients with pathologic nipple discharge or bloody nipple discharge who have no pathological findings on the imaging techniques.

## Figures and Tables

**Figure 1:**
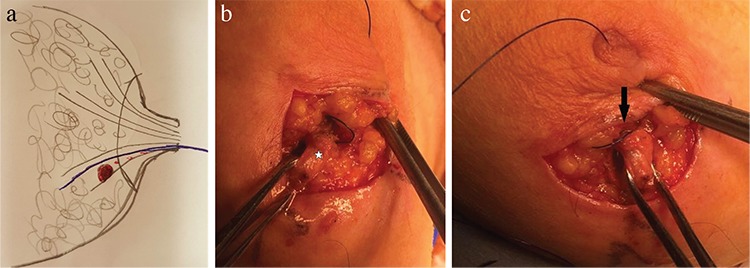
The illustration of polypropylene suture-guided microdochectomy: the duct with red spots symbolizesthe discharging duct, and the blue line shows the suture (a). The blue color of the polypropylene suture becomes noticeable throughout the pathological ductus (star) (b). Extension of the polypropylene suture from the nipple to the discharging duct can be observed; the bloody discharging duct is confirmed after observing the polypropylene after a partial incision of the duct (arrow) (c).
